# Reducing *Legionella* Colonization of Water Systems with Monochloramine

**DOI:** 10.3201/eid1204.051101

**Published:** 2006-04

**Authors:** Brendan Flannery, Lisa B. Gelling, Duc J. Vugia, June M. Weintraub, James J. Salerno, Michael J. Conroy, Valerie A. Stevens, Charles E. Rose, Matthew R. Moore, Barry S. Fields, Richard E. Besser

**Affiliations:** * Centers for Disease Control and Prevention, Atlanta, Georgia, USA;; † California Emerging Infections Program, Oakland, California, USA;; ‡ California Department of Health Services, Richmond, California, USA;; § City and County of San Francisco Department of Public Health, San Francisco, California, USA;; ¶ San Francisco Public Utilities Commission, Burlingame, California, USA

**Keywords:** Legionella, Legionnaires’ disease, water supply, disinfection, chloramines, research

## Abstract

Monochloramine disinfection of municipal water supplies is associated with decreased risk for Legionnaires' disease. We conducted a 2-year, prospective, environmental study to evaluate whether converting from chlorine to monochloramine for water disinfection would decrease *Legionella* colonization of hot water systems. Water and biofilm samples from 53 buildings were collected for *Legionella* culture during 6 intervals. Prevalence ratios (PRs) comparing *Legionella* colonization before and after monochloramine disinfection were adjusted for water system characteristics. *Legionella* colonized 60% of the hot water systems before monochloramine versus 4% after conversion (PR 0.07, 95% confidence interval 0.03–0.16). The median number of colonized sites per building decreased with monochloramine disinfection. Increased prevalence of *Legionella* colonization was associated with water heater temperatures <50°C, buildings taller than 10 stories, and interruptions in water service. Increasing use of monochloramine in water supplies throughout the United States may reduce *Legionella* transmission and incidence of Legionnaires' disease.

## Abstract 2

Monochloramine reduced colonization in building hot water systems.

Legionnaires' disease, named after an outbreak of severe pneumonia at a legionnaires' convention in 1976, is a form of community-acquired and nosocomial pneumonia. It is caused by inhalation of aerosols or microaspiration of water containing *Legionella* bacteria. *Legionella* spp. are ubiquitous in fresh water and occur naturally as intracellular parasites of amebae (1). Potable hot water systems provide environments for amplification of *Legionella pneumophila*, the most common species isolated from patients with Legionnaires' disease. *L*. *pneumophila* grows optimally at 35°C and multiplies between 25°C and 42°C. Investigations of outbreaks of Legionnaires' disease in hospitals and other community settings have implicated potable hot water systems as sources of transmission (2–5).

No strategies have been proven to prevent community-acquired Legionnaires' disease. Prevention of transmission within healthcare facilities focuses primarily on preventing or limiting *Legionella* colonization of plumbing systems through temperature control or use of biocides (6). Healthcare facilities are of special concern because of increased susceptibility to and a high case-fatality ratio of Legionnaires' disease among immunocompromised patients and those with underlying illnesses (5,7). Because colonized water distribution systems are often implicated in *Legionella* transmission (2,5,8,9), effective water disinfection strategies could provide the best measure to prevent Legionnaires' disease.

Chloramination is a method of drinking water disinfection that provides a lasting residual disinfectant in the distribution system. The process involves adding ammonia to chlorinated water; aqueous chlorine reacts with ammonia to form inorganic chloramines (10). Monochloramine is the most active compound and forms preferentially at certain ratios of ammonia to chlorine. Approximately 55% of 11.8 million people living in the 25 largest cities in California currently receive water disinfected with monochloramine (unpub. data). A survey in 2004 of municipal water utilities in the United States found that 30% used monochloramine for residual disinfection (11). The Environmental Protection Agency estimates that municipal water utilities using surface water sources will increasingly convert to monochloramine to meet federal regulations that limit disinfection byproducts in drinking water (12).

Use of monochloramine for residual disinfection compared with chlorine was associated with a lower prevalence of *Legionella* colonization in plumbing systems (13) and decreased risk of nosocomial outbreaks of Legionnaires' disease in cross-sectional and retrospective case-control studies (14,15). The planned conversion to monochloramine for municipal drinking water disinfection in San Francisco, California, provided an opportunity to prospectively investigate the effect of chloramination on *Legionella* colonization in potable hot water systems. We report here on the results of a 2-year environmental study.

## Methods

### Study Site

The San Francisco Public Utilities Commission provides an average of 250 million gallons (950 million liters) of water per day to ≈2.4 million residents in northern California, including 750,000 in the city and county of San Francisco. Surface water makes up >99% of the water supply. Chlorine was added to kill microorganisms present in source water (primary disinfection) throughout the study period. Chlorine concentrations are monitored at several locations throughout the distribution system. Chlorine used for residual (or secondary) disinfection was replaced with monochloramine on February 2, 2004.

Buildings with >3 stories in San Francisco were identified from lists of commercial customers of the San Francisco Public Utilities Commission and real property owned by the city and county of San Francisco. Building managers and owners gave permission for sample collection inside the buildings for the duration of the study. Results of *Legionella* cultures were provided only at the completion of the study. Standardized questionnaires were administered to building engineers and facilities managers to obtain information on the age of the building, capacity of water heaters and hot water storage tanks, type of water heating system (boiler, heat exchanger, or instantaneous heaters), and type of pipe material used throughout most of the building. At the completion of the study, building engineers were surveyed about routine maintenance plans for the potable hot water system, standard procedures for flushing outlets after a disruption of water service, and knowledge of industry guidelines for controlling *Legionella* growth in building water systems (16).

### Environmental Sampling

Samples from each building were collected 6 times during the 2-year period, 3 times before and 3 times after conversion to monochloramine disinfection. Each round of sampling lasted 8–10 weeks. Preconversion and postconversion rounds of sampling were conducted at corresponding seasonal intervals.

Nine samples were collected from each building during sampling rounds, including a 1-L water sample from a water heater or heat exchanger, four 1-L samples of hot water, and 4 swabs of biofilm at point-of-use outlets (faucets or shower heads). Water samples were collected in sterile, 1-L plastic bottles (Nalge Nunc International, Rochester, NY, USA) containing 0.5 mL 0.1 N sodium thiosulfate solution to neutralize free chlorine and chloramines. Water heater samples were drawn from the drain valve, pressure relief valve, or from the closest outlet to heat exchangers. Point-of-use outlets were selected at farthest points from water heaters when possible. Biofilm samples were collected from shower outlets and faucets by inserting a sterile, polyester-tipped applicator swab (Falcon, Becton Dickinson and Company, Sparks, MD, USA) and rotating it firmly against the interior surface. Biofilm swabs were placed in sterile, screw-capped test tubes containing 0.1 mL sodium thiosulfate solution in 5 mL of water from the same site. Hot water taps were run until the temperature reached a maximum for collection of water samples. The same locations were sampled in each round. When sampling could not be performed at the selected site, the nearest substitute site was sampled; however, only samples collected from the same site before and after monochloramine conversion were included in analyses.

Water temperature, pH, and free (disassociated) and total chlorine concentrations were measured in a separate sample bottle. Total chlorine includes free chlorine plus monochloramine. Temperature was measured with a hand-held thermometer. pH was measured with a digital meter (pHep 3, Hanna Instruments, Leighton Buzzard, UK). Free and total chlorine residuals were measured by using the N, N-diethyl-p-phenylenediamine method with a colorimeter and test kit (Model DR/890, Hach Chemical Co., Loveland, CO, USA). Building engineers were asked about any interruptions in water service affecting the building or specific sites in the 3 months preceding the sampling date.

### Laboratory Procedures

All culturing for *Legionella* species and amebae was performed in the Legionella Laboratory at the Centers for Disease Control and Prevention in Atlanta, Georgia, following standard procedures (17). *Legionella* organisms were speciated or serogrouped by macroscopic slide agglutination with a panel of polyclonal rabbit antisera against *Legionella* species and *L*. *pneumophila* serogroups (18). Laboratorians were blinded to the identity of buildings from which samples were obtained, and buildings were assigned different identification numbers in each round. Samples were transported at ambient temperature and processed a mean (± standard deviation) of 3 (±2) days after collection. Water samples from point-of-use outlets were concentrated 100-fold by filtration through a 0.2-μm polycarbonate filter (Nucleopore, Pleasanton, CA, USA). Biofilm swab samples were placed on a lawn of *Escherichia coli* for detection of ameba and treated with diluted acid (0.1 mol/L KCl, 0.005 mol/L HCl) to reduce the number of non-*Legionella* bacteria before plating.

Concentrations of *Legionella* spp. in water samples are expressed as CFU/mL based on plate counts of *Legionella* colonies grown from a known volume of original sample. Concentrations determined by this method are approximate. The upper and lower limits of detection were 0.05 and 25 CFU/mL for point-of-use outlets and 10 and 5,000 CFU/mL for water heaters. Plate counts were not determined for samples overgrown with non-*Legionella* organisms.

### Surveillance for Legionnaires' Disease

Active, laboratory-based surveillance for culture-confirmed *Legionella* infections in San Francisco residents was conducted from January 1, 2003, through December 31, 2004, through the Active Bacterial Core surveillance activity of the California Emerging Infections Program (19). We reviewed legionellosis case report forms from the national passive surveillance system for cases among San Francisco residents or persons with a history of travel to San Francisco during the incubation period. Surveys were sent to infection control departments at all San Francisco hospitals to identify cases of probable or confirmed Legionnaires' disease during 2003 and 2004. Information was solicited from hospitals about environmental testing for *Legionella* spp. in water systems, and measures taken to reduce microbial contamination of water systems during 2003 and 2004.

### Statistical Analysis

Data were entered into Access version 2002 (Microsoft, Redmond, WA, USA) and analyzed by using SAS for Windows version 9.0 (SAS Institute, Cary, NC, USA). We conducted building- and site-specific analyses of the prevalence of *Legionella* colonization. A building was considered colonized at a timepoint if *Legionella* spp. were cultured from any site. We considered a point-of-use outlet colonized if *Legionella* spp. were cultured from either a water sample or biofilm swab. Wilcoxon rank sum test was used to analyze differences in the proportions of positive sites or concentrations of *Legionella*. We also calculated adjusted prevalence ratios (PRs) and 95% confidence intervals (CIs) or p values by using PROC GENMOD (SAS Institute) for the clustered nature of sites within buildings. Preconversion and postconversion sampling rounds were considered repeated measures. Multivariable models investigated associations between *Legionella* colonization and water measurements or building characteristics.

## Results

### Effects of Conversion to Monochloramine on Water Distribution System

The conversion to monochloramine provided higher concentrations of total chlorine (which includes both free chlorine and monochloramine) and lower concentrations of trihalomethane compounds, the principal disinfection byproducts in treated water entering the distribution system (Table 1). The conversion to monochloramine also resulted in an ≈10-fold increase in total chlorine concentrations measured in building hot water systems. Average temperature and pH measured in building water samples did not change significantly.

**Table 1:** Characteristics of treated water in the city distribution system or in water heaters in 53 sampled buildings, San Francisco, California, stratified by year

Measurement (unit)*	2003, mean (range)	2004,† mean (range)	Reference value‡
Treated water			
Total chlorine, ppm	0.60 (0.01–2.20)	1.97 (0.15–3.40)	4.00
Total trihalomethanes, ppb	65.3 (16.0–143.0)	34.8 (11.0–46.0)	80.0
Total haloacetic acids, ppb	19.5 (6.0–55.0)	20.3 (3.0–33.0)	60.0
Total organic carbon, ppb	2.8 (2.4–3.3)	2.9 (2.6–3.1)	NA
pH	9.0 (7.4–9.9)	8.8 (7.5–10.5)	NA
Lead, ppb§	6.7§	11.5	15.0
Copper, ppb§	120§	90	1,300
Water sampled from water heaters in 53 buildings¶			
Total chlorine, ppm	0.13 (0–0.86)	1.10 (0–2.20)	NA
Temperature, °C	44.9 (17.8–87.8)	45.1 (17.8–79.4)	NA
pH	8.9 (7.5–10.5)	8.7 (7.5–10.4)	NA

*ppm, parts per million; ppb, parts per billion. †Measurements were taken from March through December 2004, after conversion to chloramine for residual disinfection. ‡Maximum allowable levels for compliance with Environmental Protection Agency standards. NA, not applicable. §Measurements for lead and copper are the 90th percentile for samples collected from point of use. Prechloramine samples were collected during 2001. ¶Water heater samples were collected on 3 occasions over a 10-month period. Mean and range were calculated for 159 samples.

### Environmental Sampling

Prospective *Legionella* testing was performed in 53 buildings, including 24 public and 29 commercial buildings. When chlorine was the residual disinfectant in municipal drinking water, *Legionella* spp. were cultured from building water systems on 96 (60%) of 159 occasions, and 37 (70%) of 53 buildings were colonized with *Legionella* spp. in >1 of the 3 sampling rounds (Table 2). After conversion to monochloramine, *Legionella* spp. were found on 7 (4%) of 159 occasions in 5 (9%) of 53 buildings. These 5 buildings had been colonized at multiple sites before disinfection with monochloramine. Conversion to monochloramine resulted in a 93% reduction in the prevalence of *Legionella* colonization in building water systems (PR 0.07, 95% CI 0.03–0.16). Colonized water systems were no more likely than *Legionella*-free systems to include hot water storage tanks or material other than copper for hot water plumbing, although sample size limited building-level analyses. *Legionella* spp. were recovered from 12 (60%) of 20 buildings for which engineers reported maintaining water systems according to standard practices, such as maintaining backflow prevention and flushing outlets after interruption of water service, versus 18 (75%) of 24 buildings for which no standard maintenance of water systems was reported (p = 0.28).

**Table 2:** Prevalence of *Legionella* colonization and concentrations in hot water systems in buildings in San Francisco, California, by residual disinfectant and sampling interval*

Water source		Chlorine (2003)			Chloramine (2004)			
		Jan–May	Jun–Jul	Oct–Dec	Mar–May	Jun–Aug	Sep–Dec	p value†
Building water systems (n = 53)								
	No. colonized with *Legionella* spp. (%)	27 (51)	34 (64)	35 (66)	5 (9)	0	2 (4)	<0.001
	Median no. *Legionella*-positive samples in colonized buildings (range)‡	3 (1–9)	3 (1–8)	4 (1–8)	1 (1–4)	–	1 (1–2)	0.007
Water heaters (n = 53)								
	No. colonized with *Legionella* spp. (%)	12 (23)	16 (30)	17 (33)	1 (2)	0	0	<0.001
	Median *Legionella* concentration in colonized water heaters, CFU/mL (range)‡	200 (40–400)	40 (10–500)	20 (10–5,000)	10	–	–	0.17
Point-of-use outlets (n = 212)								
	No. colonized with *Legionella* spp. (%)	75 (37)	85 (41)	86 (41)	7 (3)	0	2 (1)	<0.001
	Median *Legionella* concentration in colonized sites, CFU/mL (range)‡	2.5 (0.05–25.0)	1.5 (0.05–25.0)	1.0 (0.05–25.0)	0.05 (0.05–0.40)	–	0.18 (0.10–0.25)	<0.001

*Percentages calculated based on number of samples included in analyses (see Methods). †For Wilcoxon rank sum test comparing number of sites colonized with *Legionella* spp. before and after the conversion to chloramine disinfectant. ‡Includes only *Legionella*-positive sites.

A total of 364 (13%) of 2,822 water and biofilm samples yielded *Legionella* spp: 352 (25%) of 1,405 samples collected before conversion and 12 (<1%) of 1,417 samples collected after conversion to monochloramine. Five *Legionella* species and 7 serogroups of *L*. *pneumophila* were identified (Figure). *L*. *pneumophila* serogroup 1 accounted for >60% of all *Legionella* organisms. The same species of *Legionella* and serogroups of *L*. *pneumophila* were repeatedly cultured from individual sites (Figure).

**Figure:**
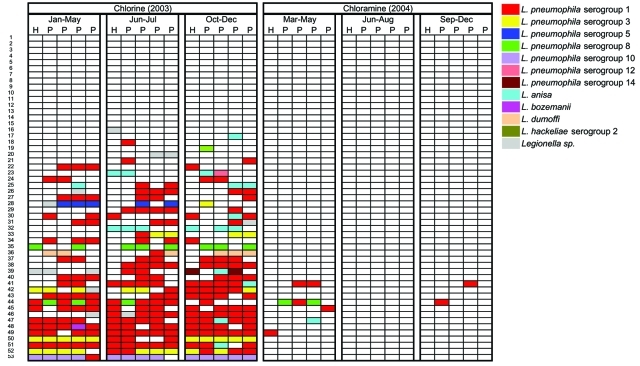
*Legionella* colonization of water heaters and point-of-use outlets sampled during 6 rounds of environmental sampling in buildings, San Francisco, California (in rows), by residual disinfectant and sampling interval. *Legionella* species or serogroups of *Legionella pneumophila* are represented with different colors. Each row represents a single building and each cell represents the results of *Legionella* culture for a site within the building. H, water heater; P, point-of-use outlet.

*Legionella* spp. were cultured from 46 (15%) of 316 water samples from building water heaters: 45 (29%) of 157 samples collected before conversion versus 1 (<1%) of 159 after conversion to monochloramine (p<0.001). When we controlled for water heater temperature, building height, and interruptions in water service, monochloramine use decreased the prevalence of *Legionella* colonization in water heaters by 96% (Table 3). Colonization of water heaters was more prevalent in buildings with >10 stories and in which water service had been interrupted in the past 3 months. Water temperatures >50°C were associated with the lowest prevalence of colonization, and *Legionella* spp. were not detected when the temperature exceeded 60°C (140°F). Over the 2-year study period, temperatures of water in building water heaters were >50°C at 88 (28%) of 318 sampling timepoints. In buildings in which engineers reported familiarity with industry guidelines for controlling *Legionella* growth in water systems, water heater temperatures were >50°C on 26 (21%) of 125 occasions versus 46 (32%) of 144 occasions in buildings in which engineers were not familiar with industry guidelines (p = 0.04).

**Table 3:** Factors associated with *Legionella* colonization of water heaters in sampled buildings, San Francisco, California

Factor	No. samples	% *Legionella* colonization	Adjusted prevalence ratio (95% CI)*	p value
Residual disinfectant				
Chlorine	157	29	Referent	
Chloramine	159	1	0.04 (0.01–0.21)	<0.001
Water heater temperature, °C				
<30	29	21	Referent	
30–39	68	24	0.43 (0.27–0.70)	0.001
40–49	131	18	0.27 (0.16–0.46)	0.001
>50	88	1	0.09 (0.05–0.18)	0.001
Building height (stories)				
3–10	215	11	Referent	
>10	101	24	2.96 (1.51–5.79)	0.002
Disruption in service in last 3 mo				
Yes	39	26	2.26 (1.31–3.88)	0.003
No	277	13	Referent	

*Prevalence ratios and 95% confidence intervals (CIs) were adjusted for repeated sampling and for effects of all other variables.

At point-of-use outlets, *Legionella* spp. were cultured from 247 (20%) of 1,252 water samples and 70 (6%) of 1,254 biofilm swab samples. Combining culture results from the water samples and biofilm swabs from each site, *Legionella* spp. were cultured from 246 (39%) of 624 paired samples before conversion to monochloramine versus 9 (1%) of 622 paired samples after conversion (p<0.001). Median concentrations of *Legionella* spp. at colonized outlets were significantly lower after conversion to monochloramine (Table 2). *Legionella* were cultured from both the water and biofilm samples on 59 (24%) of 246 occasions before conversion versus 2 (22%) of 9 occasions after conversion. The same *Legionella* species and serogroup was cultured from biofilm swabs and water samples on 56 (92%) of 61 occasions when both were positive. When we controlled for *Legionella* spp. in the sampled water heater, water temperature at the point of use, building height, and interruptions in water service, monochloramine use decreased the prevalence of *Legionella* colonization at point-of-use outlets by 96% (Table 4). *Legionella* colonization at point-of-use outlets was independently associated with *Legionella* spp. in the sampled water heater, building height, and interruptions in water service. *Legionella* spp. were cultured only from point-of-use outlets and not from water heater samples on 72 (61%) of 118 occasions when building water systems were colonized, including 6 (86%) of 7 occasions after monochloramine conversion.

**Table 4:** Factors associated with *Legionella* colonization at point-of-use outlets in sampled buildings, San Francisco, California

Factor	No. samples	% *Legionella* colonization	Adjusted prevalence ratio (95% CI)*	p value
Residual disinfectant				
Chlorine	617	39	Referent	
Chloramine	627	1	0.04 (0.02–0.09)	<0.001
Water heater colonized with *Legionella* spp.				
Yes	182	74	1.74 (1.26–2.40)	<0.001
No	1,062	11	Referent	
Temperature of water at point-of-use outlet, °C				
<30	23	13	Referent	
30–39	268	24	0.90 (0.39–2.08)	0.8
40–49	786	22	0.85 (0.35–2.05)	0.7
>50	175	10	0.75 (0.29–1.90)	0.5
Building height (stories)				
3–10	855	15	Referent	
>10	397	31	1.85 (1.46–2.35)	<0.001
Disruption in service in last 3 mo				
Yes	142	23	1.34 (0.99–1.81)	0.06
No	1,109	20	Referent	

*Prevalence ratios and 95% confidence intervals (CIs) were adjusted for repeated sampling and correlations between samples taken from the same building and for the effects of all other variables. Twenty-eight observations were missing.

Amebae at sampled sites were associated with *Legionella* spp. colonization only when chlorine was used for residual disinfection. *Legionella* spp. were cultured from 61 (36%) of 169 samples in which amebae were present versus 291 (24%) of 1,236 samples without amebae (p = 0.01). After conversion to monochloramine, *Legionella* were found in 1 (1%) of 78 samples containing amebae and 8 (1%) of 866 samples without amebae (p = 0.75). During disinfection with chlorine, *Legionella* concentration was higher in samples containing amebae (median 9.0 CFU/mL, range 0.1–25.0) compared with those without amebae (median 1.5 CFU/mL, range 0.05–25.0, p<0.001). The prevalence of amebae decreased from 169 (12%) of 1,405 samples when chlorine was the residual disinfectant to 78 (8%) of 944 samples collected in the first 2 rounds after conversion to monochloramine (p = 0.006). Results of ameba cultures from the final round of sampling were discarded after amebae were found in negative control water samples.

### Surveillance for Legionnaires' Disease

Active, population-based surveillance for *Legionella* infections identified 1 confirmed case of Legionnaires' disease in a San Francisco resident in November 2004, who traveled to Mexico within 2–10 days of symptom onset. Infection control departments in 7 (70%) of 10 hospitals in San Francisco, including the 3 largest hospitals, reported no hospitalized patients meeting the definition of a probable or confirmed case of Legionnaires' disease (20) during the study period. Review of case report forms from the national passive surveillance system did not identify any cases of Legionnaires' disease in persons with history of travel to San Francisco during the incubation period of their illness.

No environmental testing for *Legionella* spp. in hospital water systems was conducted during 2003 and 2004 in the San Francisco hospitals that responded to the survey. Two hospitals added supplemental chlorine to their water system to prevent microbial contamination before monochloramine conversion; supplemental chlorination was discontinued after monochloramine was added to municipal drinking water.

## Discussion

This is the largest study to prospectively evaluate the effect of monochloramine disinfection on *Legionella* colonization in a water distribution system. *Legionella* spp. were prevalent and stable in building water systems over 3 rounds of sampling when chlorine was used for residual disinfection of drinking water. Monochloramine disinfection of the water supply reduced *Legionella* colonization in hot water systems. Our findings suggest that monochloramine in drinking water provides better control of *Legionella* growth in building plumbing systems than chlorine. This study supports the biologic plausibility of decreased risk of nosocomial outbreaks of Legionnaires' disease associated with chloraminated water compared with chlorinated water (14,15).

The conversion from chlorine to monochloramine for residual disinfection resulted in lower concentrations of trihalomethane compounds in drinking water, which met the objectives of the municipal water supplier. Increased stability of monochloramine resulted in higher disinfectant concentrations in potable hot water systems because chlorine dissipates rapidly at higher temperatures. Higher concentrations of disinfectant and the ability of monochloramine to penetrate biofilms were likely responsible for the effect on *Legionella* spp. In model systems, monochloramine eliminates 99.9% of biofilm-associated *Legionella* spp (21), and *Legionella* spp. are cleared rapidly after addition of monochloramine (22). Although amebae in model systems protect *Legionella* spp. from the short-term effects of monochloramine (21), we found no evidence of this protective effect in the buildings we sampled.

The results of this study are relevant for strategies to control Legionnaires' disease in hospitals. Several strategies are currently used by hospitals to control *Legionella* growth in water systems and prevent nosocomial transmission of Legionnaires' disease (23). Thermal eradication (superheating water followed by flushing point-of-use outlets) and hyperchlorination were among the earliest methods effective at controlling *Legionella* growth (23,24). However, superheating increases the risk of scalding injuries and hyperchlorination is associated with increased corrosion of plumbing. Copper-silver ionization has also been used with mixed success (25–27). Monochloramine use for drinking water disinfection has been associated with lower prevalence of *Legionella* spp. in plumbing systems of hospitals (13). Our study demonstrated that *Legionella* colonization in a plumbing system was effectively eliminated by monochloramine. Hospitals or other facilities colonized with *Legionella* spp. might control *Legionella* growth and prevent disease transmission by adding monochloramine to their potable water system. The potential use of supplemental monochloramine in hospitals to prevent nosocomial Legionnaires' disease needs to be evaluated.

The results of our study are striking considering that we observed few cases of Legionnaires' disease despite evidence that *Legionella* spp. colonized most of the San Francisco buildings tested before use of monochloramine. Some cases of Legionnaires' disease may have gone undetected because patients with community-acquired pneumonia are increasingly treated empirically with antimicrobial drugs without microbiologic confirmation (28). Although we sampled 4 point-of-use outlets in each building, exposures to aerosols produced by these outlets may have been minimal. Persons exposed to any *Legionella*-containing aerosols may have been at low risk for Legionnaires' disease. Alternatively, the *Legionella* organisms present, even though some were *L*. *pneumophila* serogroup 1, might lack virulence factors needed to cause human disease (29).

Routine maintenance programs for plumbing systems were not effective in preventing colonization with *Legionella* spp., which is consistent with a previous study of hospital water systems (30). However, our findings suggest that existing guidelines were not fully implemented in the buildings sampled. Although nearly half of building engineers reported knowledge of industry guidelines for preventing *Legionella* colonization of potable water systems, only 13% of sampled water heaters were set at the recommended temperature of >60°C (140°F) (16). *Legionella* spp. were not found in water heaters set at the recommended temperature. Maintaining the recommended temperatures in water heaters could help prevent *Legionella* growth in hot water systems. Investigations of legionellosis outbreaks have consistently demonstrated that temperatures of 25°C to 42°C facilitate the growth and amplification of *Legionella* spp. to high concentrations (1).

The repeated measurement of *Legionella* colonization at the same sites over time represents a strength of this study. Colonization was stable during the first 3 sampling rounds and no seasonal effect on the prevalence of colonization was observed before conversion to monochloramine. Collection of samples at multiple point-of-use outlets in each building, in addition to water heater samples, increased detection of colonization within buildings. In an outbreak setting, widespread sampling, including sampling of sites that served as likely exposures for cases, is an important step in identifying possible sources of transmission. Filter concentration of water samples from point-of-use outlets increased the yield of positive cultures and provided additional information about the distribution of *Legionella* spp.

This study was not designed to analyze effects of conversion from chlorine to monochloramine on outcomes other than *Legionella* colonization in building water systems. Few data exist on the health effects of ingestion of monochloramine despite a long history of its use in water disinfection (31). Since monochloramine eliminates *Legionella* spp., other organisms may colonize water distribution systems (32). Our findings may be specific to characteristics of the water or distribution system in San Francisco, although they are consistent with results of a similar study in Pinellas County, Florida (33). Because monochloramine was added continuously to the municipal water supply after conversion and concentrations were maintained within specified ranges, effects on *Legionella* spp. at different monochloramine concentrations may vary.

Monochloramine disinfection of municipal water supplies is the only community-based intervention associated with reduced risk of Legionnaire's disease (14,15). Control of Legionnaires' disease is unlikely to be a major factor in a water utility's decision to convert to monochloramine for residual disinfection. However, if water suppliers increasingly convert to monochloramine to reduce concentrations of disinfection byproducts, control of the growth of *Legionella* spp. in potable water systems may be an additional health benefit.
